# Sex differences in brain activity and connectivity in late-life depression

**DOI:** 10.1093/psyrad/kkaf029

**Published:** 2025-12-01

**Authors:** Xiaomin Zheng, Ben Chen, Mingfeng Yang, Shuang Liang, Zhidai Xiao, Danyan Xu, Haoye Tan, Qiang Wang, Qin Liu, Jiafu Li, Pengbo Gao, Xiaomei Zhong, Yuping Ning

**Affiliations:** The First School of Clinical Medicine, Southern Medical University, Guangzhou, Guangdong Province, 510515, China; Geriatric Neuroscience Center, The Affiliated Brain Hospital, Guangzhou Medical University, Guangzhou, Guangdong Province, 510370, China; Geriatric Neuroscience Center, The Affiliated Brain Hospital, Guangzhou Medical University, Guangzhou, Guangdong Province, 510370, China; Geriatric Neuroscience Center, The Affiliated Brain Hospital, Guangzhou Medical University, Guangzhou, Guangdong Province, 510370, China; Geriatric Neuroscience Center, The Affiliated Brain Hospital, Guangzhou Medical University, Guangzhou, Guangdong Province, 510370, China; Geriatric Neuroscience Center, The Affiliated Brain Hospital, Guangzhou Medical University, Guangzhou, Guangdong Province, 510370, China; The First School of Clinical Medicine, Southern Medical University, Guangzhou, Guangdong Province, 510515, China; Geriatric Neuroscience Center, The Affiliated Brain Hospital, Guangzhou Medical University, Guangzhou, Guangdong Province, 510370, China; Geriatric Neuroscience Center, The Affiliated Brain Hospital, Guangzhou Medical University, Guangzhou, Guangdong Province, 510370, China; Geriatric Neuroscience Center, The Affiliated Brain Hospital, Guangzhou Medical University, Guangzhou, Guangdong Province, 510370, China; The First School of Clinical Medicine, Southern Medical University, Guangzhou, Guangdong Province, 510515, China; Geriatric Neuroscience Center, The Affiliated Brain Hospital, Guangzhou Medical University, Guangzhou, Guangdong Province, 510370, China; Geriatric Neuroscience Center, The Affiliated Brain Hospital, Guangzhou Medical University, Guangzhou, Guangdong Province, 510370, China; Geriatric Neuroscience Center, The Affiliated Brain Hospital, Guangzhou Medical University, Guangzhou, Guangdong Province, 510370, China; Geriatric Neuroscience Center, The Affiliated Brain Hospital, Guangzhou Medical University, Guangzhou, Guangdong Province, 510370, China; The First School of Clinical Medicine, Southern Medical University, Guangzhou, Guangdong Province, 510515, China; Geriatric Neuroscience Center, The Affiliated Brain Hospital, Guangzhou Medical University, Guangzhou, Guangdong Province, 510370, China; Key Laboratory of Neurogenetics and Channelopathies of Guangdong Province and the Ministry of Education of China, Guangzhou Medical University, Guangzhou, China; Guangdong Engineering Technology Research Center for Translational Medicine of Mental Disorders, Guangzhou, China

**Keywords:** late-life depression, sex difference, resting-state fMRI, regional homogeneity, amplitude of low-frequency fluctuations, cognitive impairment

## Abstract

**Background:**

There are notable sex differences in the symptoms and treatment response of late-life depression (LLD); however, the underlying static and dynamic abnormalities in brain function that may drive these disparities remain unclear. This study was to investigate sex-specific aberrant brain activity in LLD.

**Methods:**

We recruited 75 LLD patients and 164 healthy controls (HCs). Static and dynamic metrics of amplitude of low-frequency fluctuation (ALFF), regional homogeneity (ReHo), and functional connectivity (FC) were compared across four groups (LLD-female, LLD-male, HC-female, and HC-male). Correlation and moderation analyses were then used to examine whether sex moderated the associations between brain activity, cognitive impairment, and depressive symptoms.

**Results:**

First, significant interaction effects between diagnosis (LLD vs. HCs) and sex were found for ALFF in the left paracentral lobule, ReHo in the right superior temporal gyrus, and static FC (sFC) between the right superior temporal gyrus and left middle frontal gyrus. Second, in LLD-female, ReHo (right superior temporal gyrus) and sFC (right superior temporal gyrus–left middle frontal gyrus) correlated with weight, and ALFF (left paracentral lobule) correlated with visuospatial skills. Third, sex significantly moderated the relationships between ReHo (right superior temporal gyrus) and cognition, ALFF (left paracentral lobule) and depressive symptoms, and sFC (right superior temporal gyrus–left middle frontal gyrus) and depressive symptoms in the LLD group.

**Conclusion:**

Our study highlights sex differences in static brain activity related to cognitive impairment and depressive symptoms in LLD, indicating sex-specific neurobiological underpinnings for this disorder.

## Introduction

Late-life depression (LLD) has emerged as a major challenge for global public health systems, and studies indicate that the prevalence of depression begins to increase significantly at age 55, reaching approximately 2% (Kok and Reynolds, 2017). In China, the country with the fastest-ageing population worldwide, the prevalence of LLD ranges from 15% to 23%, with more than 7% of the population aged 60 years and above affected (Wen *et al*., 2022; Han *et al*., 2023a). LLD is directly associated with a 47% increased risk of dementia and a 60% increase in all-cause mortality (Zhou *et al*., 2023). Compared with younger patients with depression, elderly individuals with LLD exhibit distinct features, including chronic disease progression, high relapse rates, poor response to antidepressant medications, and unique cognitive, clinical, genetic, and neural network imaging features (Wen *et al*., 2022; Chen *et al*., 2024). While the clinical profiles highlight distinct cognitive and affective impairments in LLD patients, the underlying neurophysiological mechanisms remain poorly understood.

Functional magnetic resonance imaging (fMRI) studies have identified specific alterations in brain activity in LLD patients (Biswal *et al*., 1995; Chen *et al*., 2023), with effective connectivity within the posterior default mode network (pDMN) indicated to serve as a potential biomarker for the early prediction of LLD (Eyre *et al*., 2016; Cosío-Guirado *et al*., 2022). Compared with healthy controls (HCs), elderly depression patients show increased connectivity in the DMN, posterior superior temporal sulcus, occipital visual network, and auditory network of the right superior temporal cortex (Eyre *et al*., 2016; Zamoscik *et al*., 2018). fMRI-based predictions of treatment response in LLD patients have revealed increased functional connectivity (FC) in the left inferior frontal gyrus, triangularis, and left orbital frontal regions from baseline (Steffens *et al*., 2019). Studies have also demonstrated elevated entropy values during emotional processing in LLD patients but an abnormal reduction in the right paracentral lobule (Lin *et al*., 2019). Higher entropy values indicate greater variability and complexity, suggesting aberrant activity in the paracentral lobule region in LLD.

Notably, while sex is typically treated as a covariate in neuroimaging studies, emerging evidence has revealed interactive effects between sex and diagnosis, particularly in the temporal and parietal regions—areas where sex differences in major depressive disorder (MDD) have been consistently confirmed (Jenkins *et al*., 2018; Dorfschmidt *et al*., 2022; Mou *et al*., 2023; Wang *et al*., 2023; Li *et al*., 2024b). Investigating sex disparities in LLD holds dual significance. The female-predominant incidence reflects distinct neurobiological pathways shaped by aging, which drive sex-specific neuroadaptive responses to stress. Clinically, women report more somatic symptoms (e.g. pain), whereas men exhibit greater cognitive decline and agitation, contributing to diagnostic inaccuracies—particularly male underdiagnosis (Li *et al*., 2024). Our team has made progress in exploring sex differences in brain activity in LLD patients, and a recent study revealed sex-specific aberrant functional connectivity of the habenula in LLD (Su *et al*., 2025). However, the sex-specific patterns of whole-brain regional spontaneous activity (encompassing both static and dynamic metrics) remain largely unexplored.

Amplitude of low-frequency fluctuations (ALFF), which measures neural activity intensity, and regional homogeneity (ReHo), which assesses local synchronization, are widely used to quantify intrinsic spontaneous neural activity independent of specific tasks (Chen *et al*., 1999; Song *et al*., 2014; Huang *et al*., 2017; Guo *et al*., 2024). Growing evidence extends their utility beyond static metrics to include dynamic ALFF (dALFF) and dynamic ReHo (dReHo), which capture time-varying neural dynamics. Substantial evidence confirms widespread abnormalities in ALFF and ReHo across depressive populations (spanning age groups), particularly involving prefrontal, limbic, and default mode network regions (Li *et al*., 2018; Liu *et al*., 2021). Notably, such alterations are not unique to depression: previous studies have documented deviations in dynamic brain activity metrics (e.g. dALFF and dReHo) that have also been reported in neuropsychiatric conditions, including amyotrophic lateral sclerosis, schizophrenia, anxiety, and insomnia (Wang *et al*., 2019a; Ma *et al*., 2020; Guo *et al*., 2024; Li *et al*., 2024), where these indices not only differ from those of HCs but also correlate with cognitive impairment (Wang *et al*., 2019b,c; Fu *et al*., 2021). In depression, these static and dynamic metrics are significantly associated with core symptoms (e.g. depressed mood, anhedonia, and cognitive impairment) and display sex-specific patterns—particularly heightened temporal–parietal and frontoparietal abnormalities in males (Jenkins *et al*., 2018; Dorfschmidt *et al*., 2022; Wang *et al*., 2023). Collectively, these findings establish ALFF and related metrics (e.g. ReHo) as effective for detecting diagnosis-by-sex interactions, enabling the identification of differences in intrinsic neural activity between male and female brains and their distinct interactions with depressive mechanisms.

The aim of this study was to explore how sex differences in ALFF, ReHo, and FC are related to clinical symptoms and cognitive impairment in patients with LLD. To test this hypothesis, we investigated (i) sex-stratified abnormalities in ALFF, ReHo, and seed-based FC, and (ii) quantitative relationships between aberrant brain metrics and sex-specific symptom–cognitive domains.

## Methods

### Participants

In this study, clinical data from 239 participants (aged ≥55 years) were utilized. The participants included 75 individuals meeting DSM-IV criteria for LLD and 164 demographically matched controls with no lifetime history of affective disorders. The specific inclusion and exclusion criteria are detailed in the supplementary information. The research protocol received ethical approval from the Institutional Review Board of Guangzhou Medical University Affiliated Brain Hospital (approval number: 2014-078). Diagnostic ascertainment followed a dual-rater system in which board-certified psychiatrists independently evaluated participants using structured clinical interviews. Participants then underwent a comprehensive assessment, which included clinical evaluations, neuropsychological tests, and structural and resting-state fMRI (rsfMRI) scans.

### Clinical assessments

Subjects in the LLD and HC groups were requested to rate the severity of their depressive symptoms using the clinician assessment scale—the 17-item Hamilton Depression Rating Scale (HAMD-17) (Schwab *et al*., 1967). The scale’s symptom dimensions were categorized into the following five factors (Zhao *et al*., 2019). (i) The anxiety/somatization score corresponded to mental anxiety (Item 10), somatic anxiety (Item 11), gastrointestinal symptoms (Item 12), general somatic symptoms (Item 13), hypochondriasis (Item 15), and insight (acknowledgement/denying being ill, Item 17). (ii) The weight score was equivalent to weight loss (Item 16). (iii) The cognitive bias score was equivalent to feelings of guilt (Item 2), suicide (Item 3), and agitated behaviour (Item 9). (iv) The retardation score was equivalent to depressed mood (Item 1), loss of interest in hobbies or work (Item 7), slowness of thought and speech, impaired ability to concentrate, decreased initiative (Item 8), and sexual symptoms (Item 14). (v) The sleep disturbance score corresponded to difficulty falling asleep (Item 4), light sleep (Item 5), and early wakefulness (Item 6).

### Cognitive function

Global cognitive function was assessed in all participants through the Mini-Mental State Examination (MMSE) (Tombaugh and McIntyre, 1992), administered by neuropsychologists. Additionally, a comprehensive neuropsychological battery was used to evaluate the following five cognitive domains: (i) memory: Auditory Verbal Learning Test (AVLT) (Q. H. Guo, 2007) and Working Memory Test (WMT) (Iverson, 2001); (ii) information processing speed: Symbol-Digit Modality Test (SDMT) (Fellows and Schmitter-Edgecombe, 2019), Stroop Color and Word Test Part A (Stroop A) (Jensen and Rohwer, 1966) and Trail-Making Test Part A (TMT A) (Tombaugh, 2004); (iii) executive function: Stroop Color and Word Test Part B (Stroop B) (Jensen and Rohwer, 1966) and Trail-Making Test Part B (TMT B) (Tombaugh, 2004); (iv) language: Boston Naming Test (BNT) (Bezdicek *et al*., 2021) and Verbal Fluency Test (VFT) (Troyer *et al*., 1997); and (v) visuospatial skills: Rey–Osterrieth Complex Figure Test (ROCF) (Hawkins *et al*., 2004).

### Magnetic resonance imaging data acquisition

Following neuropsychological assessments, participants underwent MRI scanning using a Philips Achieva 3.0T system (Netherlands) at the Affiliated Brain Hospital of Guangzhou Medical University. The imaging protocol comprised two components: (i) a high-resolution 3D T1-weighted anatomical scan with gradient echo acquisition and (ii) an 8-min rsfMRI session employing a single-shot gradient echo-planar imaging sequence. Key parameters for the rsfMRI included an echo time (TE) of 30 ms, repetition time (TR) of 2000 ms, flip angle of 90°, 33 slices (4 mm thickness), 64 × 64 matrix, and 220 × 220 mm field of view.

### Magnetic resonance imaging data preprocessing

Preprocessing of the rsfMRI data was performed using the Data Processing Assistant for Resting-State fMRI (DPABI 4.2) based on Statistical Parametric Mapping (SPM12). The first 10 volumes were discarded to allow for magnetic field stabilization. The remaining images underwent slice-timing correction, realignment for head motion correction, and calculation of head motion parameters. Participants with >2 mm maximum displacement, >2° rotation, or >0.2 mm mean framewise displacement were excluded. The realigned images were then spatially normalized to the Montreal Neurological Institute (MNI) echo-planar imaging template, resampled to 3 × 3 × 3 mm^3^ voxels, and linearly detrended. A bandpass filter (0.01–0.1 Hz) was applied to reduce low-frequency drift and high-frequency noise. Nuisance signals from white matter, cerebrospinal fluid, and Friston-24 head motion parameters were regressed out.

### Analysis of ALFF and ReHo

For static metrics, rsfMRI data (without bandpass filtering) were processed to compute ALFF and ReHo. ALFF was derived by transforming each voxel’s BOLD (blood oxygen level-dependent) time series to the frequency domain via Fast Fourier Transform (FFT) and extracting the square root of the mean power in the 0.01–0.1 Hz band. ReHo was calculated using Kendall’s coefficient of concordance (KCC) to quantify temporal synchronization between each voxel and its 26 nearest neighbours.

Dynamic metrics (dALFF and dReHo) were analysed using a sliding-window approach with a 50-TR Hamming window and 1-TR step size to capture transient fluctuations. Analyses were repeated with window sizes of 30 and 70 to stabilize the results. For dALFF, the time series were segmented into overlapping windows; for dReHo, unsmoothed data were similarly segmented. Window-specific KCC values were concatenated to generate whole-brain dALFF and dReHo time series.

Both static and dynamic metrics were normalized to Z scores (voxelwise mean divided by global mean). ALFF maps were analysed without prior spatial smoothing to avoid high-frequency noise contamination. ReHo maps were spatially smoothed with a 6 mm full width half-maximum (FWHM) Gaussian kernel (to increase the group-level signal-to-noise ratio).

Voxelwise among-group comparisons (LLD-male, LLD-female, HC-male, and HC-female) were conducted to identify regions with significant ALFF and ReHo differences [voxel *P* < 0.001, cluster *P* < 0.05, Gaussian random field (GRF)-corrected]. Masks were generated using XjView, and ALFF and ReHo values were extracted via DPABI_V4.2 for subsequent statistical analyses.

### Region of interest definition and analysis of static functional connectivity

Clusters of ReHo and ALFF that appeared in group differences were used as regions of interest (ROIs) for the FC analysis. Static FC (sFC) was examined using a seed-based whole-brain approach. Individual sFC maps were generated by calculating Pearson correlation coefficients between the time series of the ROIs and all other voxels. Maps were transformed to z maps via Fisher’s r-to-z transformation and smoothed with a 6-mm FWHM Gaussian kernel. Finally, interaction analyses including age, years of education, and head motion as covariates were used to compare FC between LLD-male, LLD-female, HC-male, and HC-female groups. Significance was determined using a cluster-level corrected threshold of *P* < 0.05 (cluster-forming threshold at voxel level *P* < 0.001 using the GRF method). Next, post-hoc two-sample *t* tests examined the differences in mean FC between groups in the brain regions identified using the previous analysis of covariance (ANCOVA). Multiple comparison correction was performed using the Bonferroni method, with significance set at *P* < 0.05.

### Statistical analysis

Statistical analyses were performed using SPSS 26.0 (SPSS, Chicago, IL, USA). The differences in demographic information between the LLD-male group, LLD-female group, HC-male group, and HC-female group were assessed using one-way ANOVA. Neuropsychological scores among the four groups were evaluated using a Kruskal‒Wallis test. Partial correlations were conducted to investigate the associations between imaging metrics (ReHo, ALFF, and sFC) and the neuropsychological scores as well as the depressive symptoms, and age and years of education were used as covariates. The results of the correlation analysis were corrected using the Benjamini method at a significance level of *P *< 0.05. For cluster-level multiple comparison correction, GRF was used (voxel *P* < 0.001; cluster *P* < 0.05). Two-way ANOVA with sex (male vs. female) and depression status (depressed vs. control) was subsequently used to examine the interaction effects of cognitive scores, HAMD-17 scores, and ReHo, ALFF, and sFC in the four groups.

Moderation analyses were conducted to investigate the potential moderating effects of sex on the relationships between depressive factors, neuropsychological scores, and ReHo, ALFF, and sFC in LLD patients. The moderation model included regional brain activity (ALFF, ReHo, and sFC) as predictors, sex as the moderator, and depressive factors and neuropsychological scores as the outcome variables, with age and years of education as covariates.

## Results

### Demographic characteristics and clinical data

The demographic characteristics and clinical data of the participants are listed in Table 1. There were no significant differences in age or years of education among the four groups (*P *> 0.05).

**Table 1:** Demographic data and clinical characteristics between LLD patients and HCs.

	LLD		HC					
	Male (*n* = 27)	Female (*n* = 48)	Male (*n* = 55)	Female (*n* = 109)	*P*	Diagnosis status F(*P*)^c^	Sex status F(*P*)^c^	Diagnosis×sex F(*P*)^c^
Age (years)	67.67 ± 7.49	66.33 ± 6.41	68.29 ± 6.95	67.04 ± 6.05	0.424^a^	0.488(0.485)	1.855(0.174)	0.002(0.967)
Years of education	10.06 ± 3.73	8.85 ± 3.32	10.19 ± 3.68	10.13 ± 3.18	0.096^a^	2.039(0.155)	1.616(0.205)	1.333(0.249)
**Cognitive functions**								
**Global cognitive function**								
MMSE	25.5(5)	26(4)	27(3)	27(3)	0.002* ^b^	11.753(0.001**)	0.395(0.53)	1.749(0.188)
**Memory**								
AVLT (N4)	5(4)	6(3)	5.5(4)	7(3)	0.03* ^b^	10.364(0.001**)	2.809(0.095)	0.121(0.728)
WMT	4(3)	5(4)	6(4)	6(4)	0.027* ^b^	5.908(0.0168*)	0.102(0.75)	0.079(0.778)
**Information processing speed**								
SDMT (s)	29(11)	27(20)	32(17)	35(14)	<0.001*** ^b^	**12.776(**<0.001***)	0.308(0.58)	0.555(0.457)
TMT A	57(27)	67(34)	47(28)	48(25)	0.006* ^b^	10.524(0.001**)	2.076(0.151)	0.455(0.501)
Stroop A (s)	33(10)	31(8)	30 (12)	27 (8)	0.001** ^b^	4.854(0.029*)	8.545(0.004**)	0.105(0.746)
**Executive function**								
TMT B	91.56 ± 70.48	92.8 ± 47.27	77.93 ± 35.23	86.67 ± 51.82	0.47^a^	2.479(0.117)	1.393(0.239)	0.507(0.477)
Stroop B (s)	47.5(16)	40(14)	43.5(21)	37(11)	0.007* ^b^	2.03(0.156)	9.134(0.003**)	0.151(0.698)
**Language**								
BNT	22(5)	20(3)	23(5)	21(4)	<0.001*** ^b^	6.275(0.013*)	7.883(0.005**)	1.318(0.252)
VFT	12(2.75)	13(6)	13(5.25)	14(5.5)	0.019* ^b^	2.77(0.098)	6.241(0.013*)	0.117(0.732)
**Visuospatial skill**								
ROCF	8(3.88)	7(9.5)	11.25(8.5)	10.5(9)	0.015* ^b^	5.264(0.023*)	1.417(0.235)	0.003(0.959)
**HAMD-17 factors**								
Retardation	2(4)	2(4)	0(0)	0(0)	<0.001*** ^b^	104.048(<0.001***)	1.098(0.296)	0.166(0.684)
Cognitive bias	1(2)	0(1.75)	0(0)	0(0)	<0.001*** ^b^	50.371(<0.001***)	0.012(0.915)	<0.001(0.983)
Anxiety/somatization	2(3)	3(5)	0(1)	0(2)	<0.001*** ^b^	83.39(<0.001***)	1.379(0.241)	1.93(0.166)
Sleep disturbance	2(4)	2(3)	0(1)	0(2)	<0.001*** ^b^	27.563(<0.001***)	3.448(0.065)	0.198(0.656)
Weight	0(0)	0(0)	0(0)	0(0)	<0.001*** ^b^	22.824(<0.001***)	0.942(0.333)	0.625(0.43)
total score	7(11)	7.5(12)	1(3)	1(3)	<0.001*** ^b^	122.046(<0.001***)	0.41(0.523)	0.02(0.889)

Note: 's' denotes seconds, indicating the time taken to complete these tests.

One-way ANOVA was used to compare the age, years of education, and TMT B between diagnosis and sex. When controlled for age and years of education, four groups (LLD-female, LLD-male, HC-female, and HC-male) were compared using a general liner model (multivariate), showing that there are significant differences in diagnosis status and sex status of cognitive functions, while there was no significant difference of depressive symptoms between the four groups in sex status and diagnosis×sex status.

a One-way ANOVA, with data expressed as mean ± SD.

b Kruskal–Wallis test, with data expressed as median (interquartile range).

c General linear model.

Abbreviations:

MMSE, Mini-Mental State Examination; AVLT(N4), Short Delay Free Recall of Auditory Verbal Learning Test; WMT, Working Memory Test;

SDMT, Symbol Digit Modalities Test; Stroop, Stroop Color and Word Test; TMT, Trail Making Test; BNT, Boston Naming Test; VFT, Verbal Fluency Test; ROCF, Rey-Osterrieth Complex Figure; LLD, late-life depression; HCs, healthy controls; HAMD-17, 17-item Hamilton Depression Rating Scale.

*P < 0.05, **P < 0.01, ***P < 0.001.

### Comparison of depressive symptoms and cognitive functions among the four groups

There were significant differences in depressive symptoms among the four groups, while no significant differences were observed between the LLD-male and LLD-female groups.

There were also significant differences in cognitive function among the four groups (MMSE, AVLT, WMT, TMT A, Stroop A, Stroop B, BNT, VFT, and ROCF). Several cognitive scores, including TMT A and TMT B, were higher in the LLD group than in the HC group. Conversely, other cognitive scores, namely MMSE, SDMT, DST, AVLT, WMT, BNT, VFT, and ROCF, were lower in the LLD group than in the HC group. Additionally, a univariate general linear model was used to examine group differences (LLD vs. HCs) and sex effects across individuals with different levels of cognitive impairment. Main effects of diagnosis were observed for MMSE [*F*(1 235) = 11.753, *P *= 0.001], AVLT [*F*(1 235) = 10.364, *P *= 0.001], WMT [*F*(1 235) = 5.908, *P *= 0.0168], SDMT [*F*(1 235) = 12.776, *P *< 0.001], TMT A [*F*(1 235) = 10.524, *P *= 0.001], Stroop A [*F*(1 235) = 4.854, *P = *0.029), BNT [*F*(1 235) = 6.275, *P *= 0.013], and ROCF [*F*(1 235) = 5.264, *P *= 0.023]. Main effects of sex emerged for Stroop A [*F*(1 235) = 8.545, *P = *0.004], Stroop B [*F*(1 235) = 9.134, *P = *0.003], BNT [*F*(1 235) = 7.883, *P *= 0.005], and VFT [*F*(1 235) = 6.241, *P *= 0.013]. No significant interactions between diagnosis and sex were detected for cognitive function (Table 1).

### ALFF and ReHo analysis in patients with LLD and HCs

**Figure 1:**
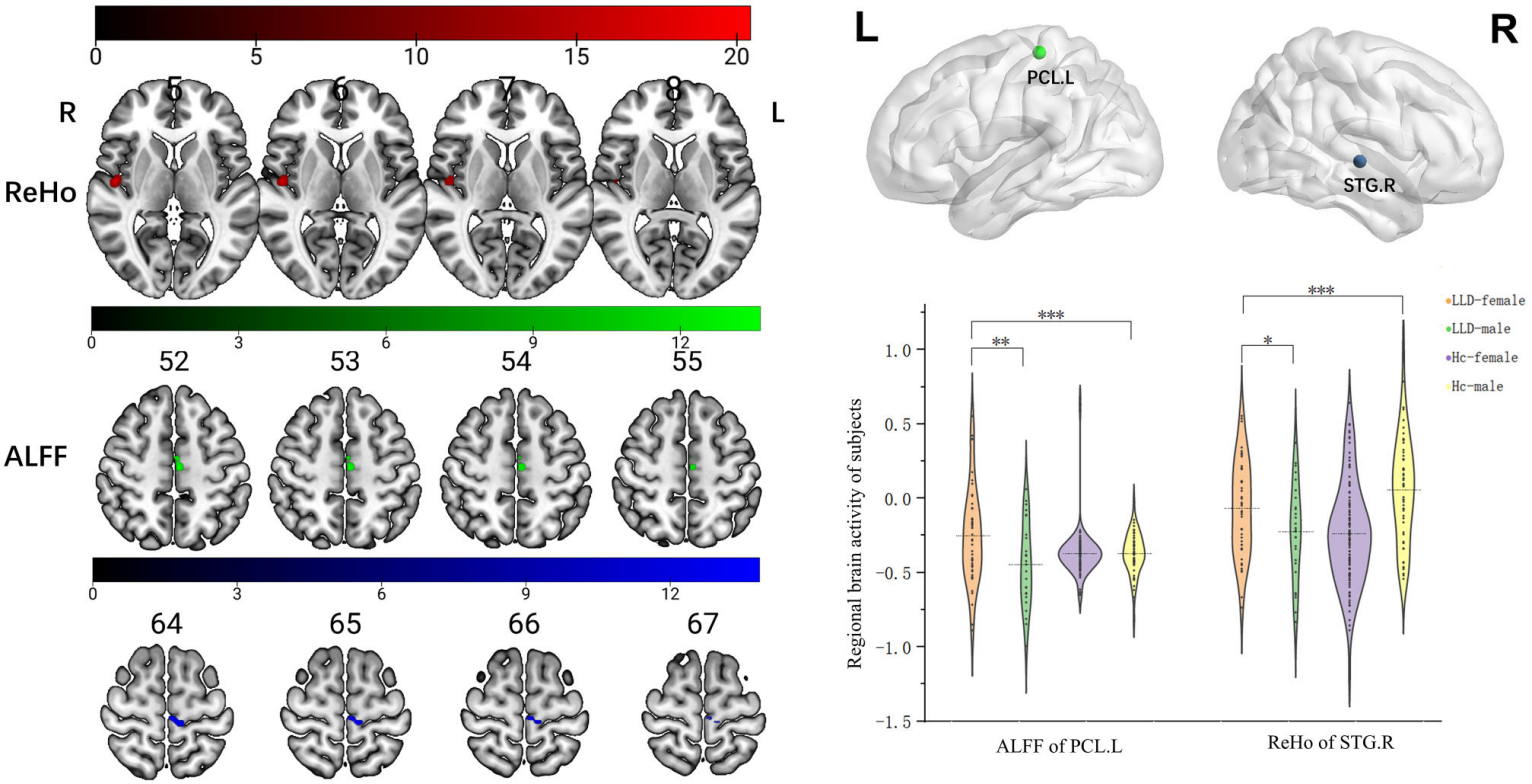
ALFF and ReHo analyses in patients with LLD and HCs. The results showed that both ALFF of left paracentral lobule and ReHo of right superior temporal gyrus had interactive effects between diagnosis and sex (tests of between-subjects effect in diagnosis×sex: *P* = 0.001, *P* < 0.001; effect size = 11.018, 22.073). In addition, an independent samples *t*-test indicated a statistically significant difference in ReHo and ALFF between LLD-female and LLD-male, suggesting sex-specific alteration in regional brain activity among individuals with late-life depression (covariant: age, years of education, and head motion). Abbreviations: ALFF, amplitude of low-frequency fluctuations; ReHo, regional homogeneity; PCL.L, left paracentral lobule; STG.R, right superior temporal gyrus. *P < 0.05, **P < 0.01, ***P < 0.001.

There was a statistically significant difference in ALFF values in the left paracentral lobule (peak MNI: −12, −30, 63; cluster size: 16) among the four groups (GRF correction, *P *< 0.05). A univariate general linear model was used to examine group differences (LLD vs. HC) and sex effects across ALFF in the left paracentral lobule. There was a main effect of sex and an interaction effect between diagnosis and sex on ALFF values in this region. For ReHo, there was a statistically significant difference in the right superior temporal gyrus (peak MNI: 51, −15, 0; cluster size: 51) among the four groups (GRF correction, *P *< 0.05). A univariate general linear model was used to examine group differences (LLD vs. HC) and sex effects on ReHo in this region. An interaction effect between diagnosis and sex was detected (Fig. 1, Table 2; Supplementary Material: Table 1).

**Table 2:** The interactive effect value of ALFF and ReHo.

	Brain region	Peak MNI			Cluster size	peak *F*
		X	Y	Z		
ALFF	Left paracentral lobule	−12	−30	63	16	14.34
ReHo	Right superior temporal gyrus	51	−15	0	51	20.87
sFC	Right superior temporal gyrus–left middle frontal	−21	27	51	43	19.79

MNI, Montreal Neurological Institute; X, Y, Z are the coordinates of primary peak locations in the MNI space; *P* < 0.05, GRF-corrected. We regressed age, years of education, and head motion as covariance by using interaction analysis.

Abbreviations:

ALFF, amplitude of low-frequency fluctuations; ReHo, regional homogeneity; sFC, static functional connectivity.

Dynamic analysis revealed significant interactions between diagnosis and sex at window sizes of 30, 50, and 70. However, there were no overlapping brain regions at window sizes of 30 and 50, except at window sizes of 30 and 70, suggesting that the dynamic results were not representative (Supplementary Material: Tables 2–4).

### ReHo-based sFC analysis in patients with LLD and HCs

A seed-to-voxel sFC analysis was conducted using the ReHo and ALFF results as seeds. A statistically significant difference in sFC was found between the ReHo of the right superior temporal gyrus and the left middle frontal gyrus (peak MNI: −21, 27, 51; cluster size: 43) among the four groups (GRF correction, *P *< 0.05). In contrast, no significant sFC was detected by the ALFF-based method.

**Figure 2:**
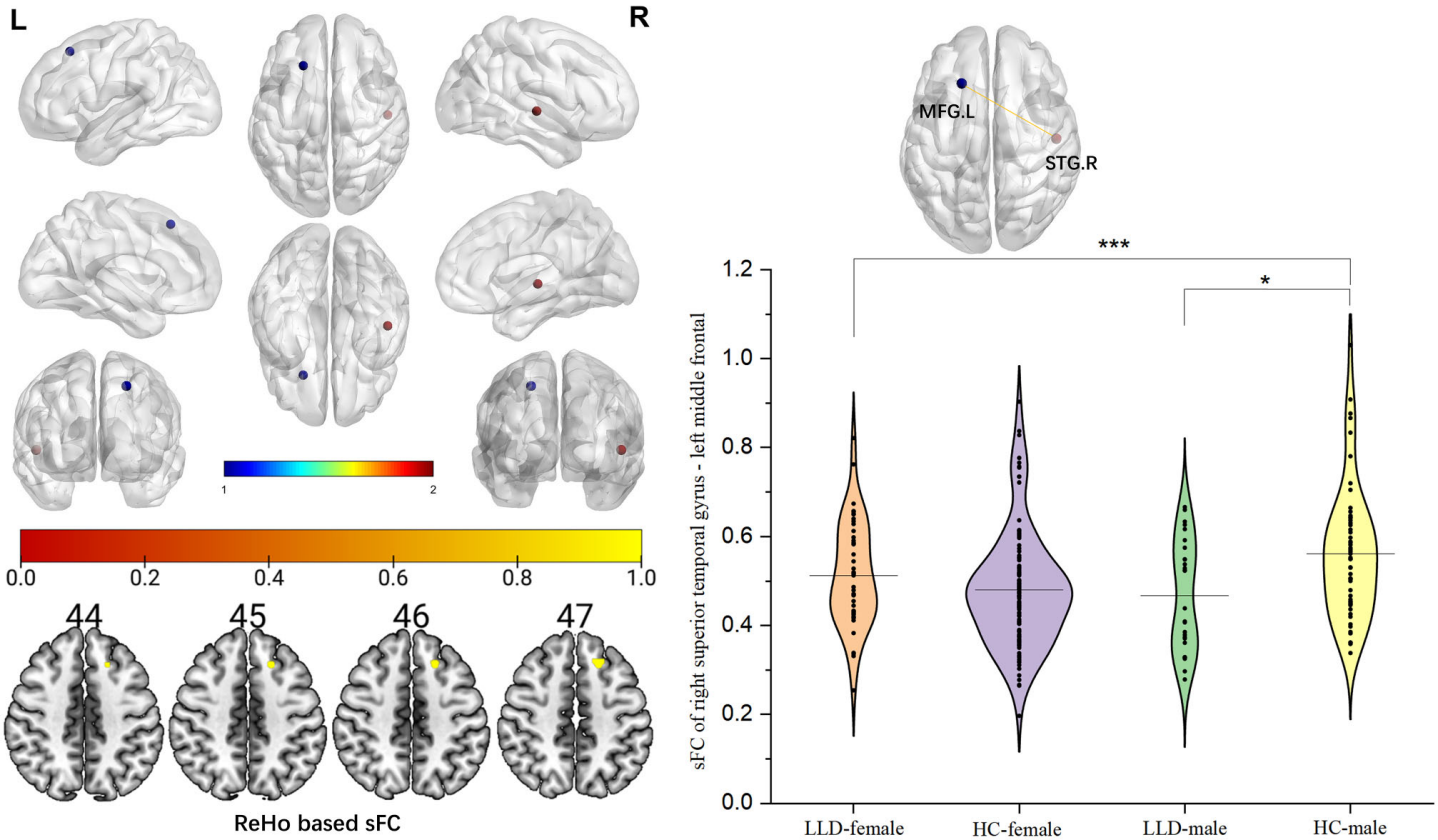
sFC analysis in patients with LLD and HCs. The results showed that sFC of ReHo of right superior temporal gyrus–left middle frontal had interactive effects between diagnosis and sex (tests of between-subjects effect in diagnosis×sex; *P* < 0.001, effect size = 10.994). In addition, an independent samples *t*-test indicated a statistically significant difference in sFC between LLD-male and HC-male groups (*P* = 0.015, effect size = 6.244), suggesting disease-specific alteration in regional brain activity among individuals with sex difference (covariant: age, years of education, and head motion). Abbreviations: ReHo, regional homogeneity; sFC, static functional connectivity; STG.R, right superior temporal gyrus; MFG.L, left middle frontal gyrus. *P < 0.05, **P < 0.01, ***P < 0.001.

A univariate general linear model was then used to examine group differences (LLD vs. HC) and sex effects across sFC between the right superior temporal gyrus and left middle frontal gyrus. There was an interaction effect of diagnosis and sex on sFC between the right superior temporal gyrus and the left middle frontal gyrus (Table 2; Supplementary Material: Table 1). Additionally, pairwise comparisons revealed a marked group difference between LLD-male and HC-male: compared with HC-male, LLD-male exhibited significantly lower sFC variability values, indicating reduced variability in functional coupling strength between these regions (Fig. 2).

### Correlation analysis between ALFF, ReHo, and sFC of brain regions and HAMD-17 factors and cognitive function in LLD patients

**Figure 3:**
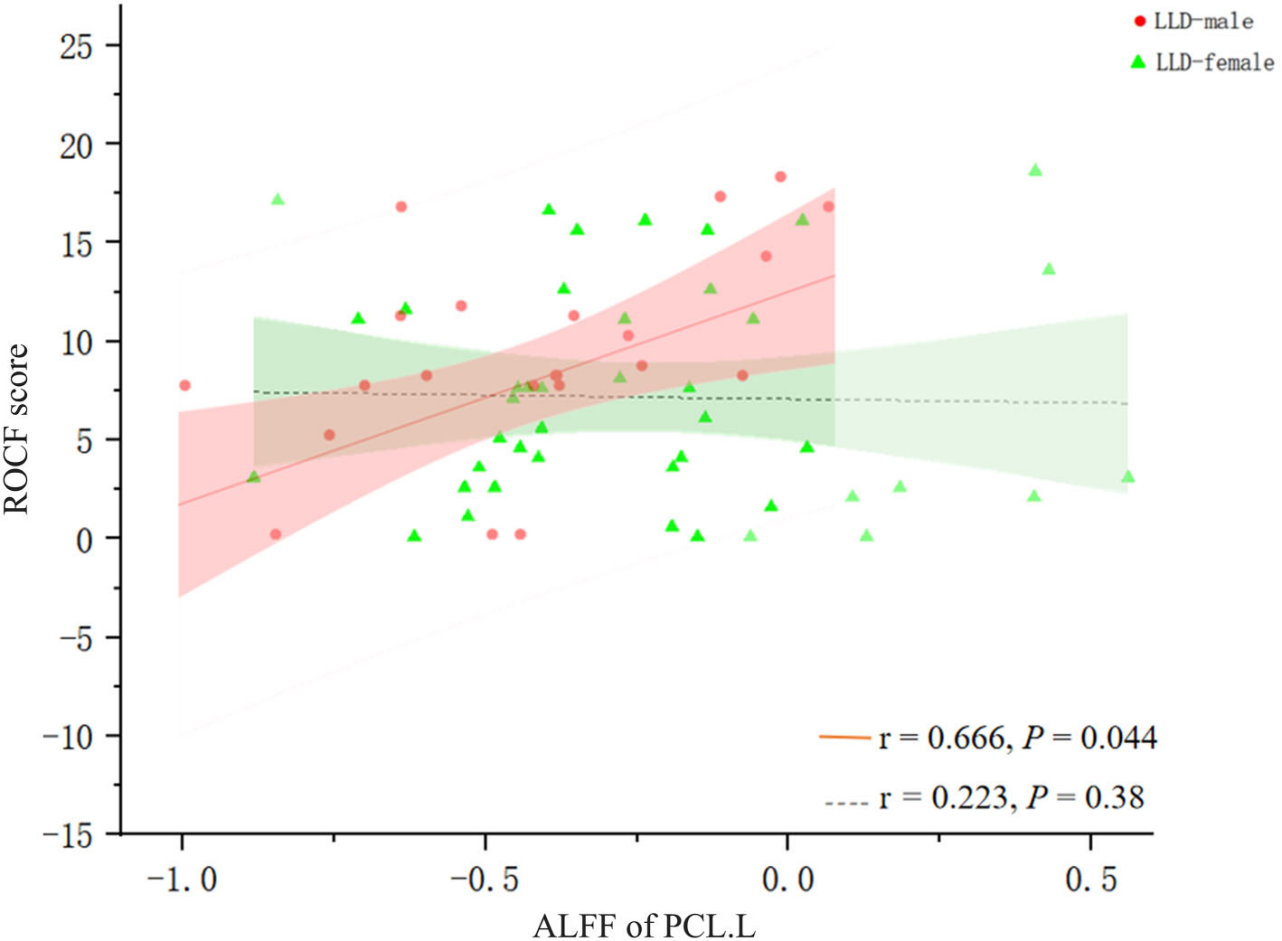
Partial correlation analysis between ROCF score and ALFF of left paracentral lobule. There was partial correlation between ROCF and ALFF of left paracentral lobule in LLD-male [*r* = 0.666, *P* = 0.044 (Benjamini corrected)], but not in LLD-female [*r* = 0.223, *P* = 0.38 (Benjamini corrected)] (age, years of education, and head motion as covariant). Abbreviations: ROCF, Rey-Osterrieth Complex Figure; ALFF, amplitude of low-frequency fluctuations; PCL.L, left paracentral lobule.

In the LLD-female group, the ReHo of the right superior temporal gyrus was positively correlated with the HAMD-17 score (weight) (*r* = 0.392; *P = *0.008; corrected *P = *0.04), and ReHo-based sFC was positively associated with HAMD-17 score (weight) (*r* = 0.436; *P = *0.003; corrected *P = *0.02). In the LLD-male group, the ALFF of the left paracentral lobule was positively correlated with the ROCF score (*r* = 0.666, *P *= 0.004, corrected *P *= 0.044) (Fig. 3; Supplementary Material: Table 5). No significant correlations were found between ALFF and HAMD-17 or between ReHo and sFC with cognitive function in either sex group.

### Moderation effects driven by sex

We examined the interaction effects of sex and ReHo on cognitive function, sex and ALFF, sFC on depressive symptoms.

In the LLD group, when ReHo served as a predictor variable, MMSE score was significantly correlated with sex (β = −3.015; *P *< 0.001) and ReHo (β = 0.736; *P =* 0.01). Additionally, there was an interaction effect between ReHo and sex (β = −4.624; *P = *0.021) on MMSE scores (*R*^2^ = 0.402; *P *< 0.001). When ALFF served as a predictor variable, depressive symptom weight was significantly correlated with sex (β = 0.384; *P = *0.001), and there was a significant interaction between sex and ALFF (β = 0.863; *P = *0.016) on weight (*R*^2 ^= 0.196; *P *< 0.05). When sFC served as a predictor variable, a significant interaction effect was observed between sex and sFC (β = −2.695; *P = *0.023; *R*^2 ^= 0.118; *P *< 0.05) on weight (Table 3; Supplemental Material: Tables 6–8, Figs 1 and 2).

**Table 3:** Sex acts as a moderator in the relationship between ALFF (left paracentral lobule), ReHo (right superior temporal gyrus), sFC (right superior temporal gyrus–left middle frontal), and depressive symptoms and cognitive function in LLD.

Model	Estimate	SE	*T*	*P*
**Model 1: Weight (HAMD-17 factor)**				
Intercept	−0.159	0.511	−0.311	0.757
ALFF of left paracentral lobule	−0.061	0.19	−0.321	0.75
Sex	0.384	0.107	3.605	0.001**
ALFF of left paracentral lobule×sex	0.863	0.349	2.477	0.016*
**Model 2: MMSE**				
Intercept	7.366	3.123	2.359	0.531
ReHo of right superior temporal gyrus	0.736	1.169	0.629	0.010*
Sex	−3.015	0.64	−4.711	<0.001***
ReHo of right superior temporal gyrus×sex	−4.624	1.951	−2.37	0.021*
**Model 3: Weight (HAMD-17 factor)**				
Intercept	1.949	0.727	2.679	0.009*
sFC of right superior temporal gyrus and left middle frontal	0.117	0.067	1.751	0.084
Sex	0.051	0.142	0.358	0.721
sFC of right superior temporal gyrus and left middle frontal×sex	−2.695	1.157	−2.330	0.023*

Abbreviations: HAMD-17, 17-item Hamilton Depression Rating Scale; MMSE, Mini-Mental State Examination; ALFF, amplitude of low-frequency fluctuations; ReHo, regional homogeneity; sFC, static functional connectivity.

*P < 0.05, **P < 0.01, ***P < 0.001.

## Discussion

This study revealed significant sex differences in resting-state brain activity among LLD patients using static fMRI metrics (ALFF, ReHo, and FC). In contrast, dynamic measures (dALFF and dReHo) showed no stable diagnostic–sex interactions. Our core findings revealed three points of sexual dimorphism: static ALFF in the left paracentral lobule (PCL.L), static ReHo in the right superior temporal gyrus (STG.R), and sFC of the STG.R and left middle frontal gyrus (MFG.L). The dissociation between static and dynamic metrics suggests that sex influences neural activity differently depending on the temporal scale measured. Specifically, the observed static alterations in ALFF and ReHo may reflect more stable or trait-like neurobiological differences associated with chronic adaptations in LLD patients. In contrast, preserved dynamic fluctuations could indicate that temporal flexibility was maintained despite the presence of pathology, as suggested by previous research (Ingalhalikar *et al*., 2014; Tavor *et al*., 2016; Gal *et al*., 2022).

ALFF quantifies spontaneous low-frequency BOLD signal amplitude (Chen *et al*., 1999; Yao *et al*., 2014a; Huang *et al*., 2017). We extended previous research linking paracentral lobule abnormalities to somatic symptoms in depression (Yao *et al*., 2014a; Liu *et al*., 2021) by identifying a male-specific pattern. In male LLD patients, higher ALFF in the PCL.L was significantly positively correlated with better visuospatial performance (ROCF *r* = 0.666, corrected *P *= 0.044). This association suggests a potential link between increased low-frequency activity in this sensorimotor hub (Patra *et al*., 2022) and preserved visuospatial function in males with LLD. Conversely, elevated ReHo in the STG.R was correlated with depressive symptoms in female, consistent with established sex differences in temporal lobe function and its role in auditory–emotional integration (Dai *et al*., 2012; Jung *et al*., 2015; Xu *et al*., 2015; Wang *et al*., 2019c; Tu *et al*., 2022). While dALFF fluctuations typically indicate cognitive flexibility (Liao *et al*., 2019) and dReHo reflects neural adaptability (Deng *et al*., 2016), the instability of sex interactions parallels previous reports in hippocampal subregions (Wang *et al*., 2015; Chen *et al*., 2023). Measures such as ALFF and ReHo reflect steady-state neural properties across time. Their calculation is not parameter-dependent, yielding reproducible results suitable for detecting stable sex moderation effects (Yan *et al*., 2016; Sbaihat *et al*., 2022). Dynamic analysis (e.g. dFC and dReHo) depends heavily on window size choice (Hutchison *et al*., 2013). This inherent instability makes reliably detecting sex‒disease interactions difficult. These findings indicate that the temporal dynamics of these metrics may be less sensitive to sex-specific pathology in LLD patients at this stage or that fundamental temporal flexibility was preserved.

Functional connectivity analysis further highlighted these divergent pathways. Using STG.R and PCL.L as ROIs, we observed a pivotal disease‒sex interaction in the sFC of the right temporal superior gyrus–left middle frontal (STG.R–MFG.L) pathway, with a significant group difference between LLD-male and HC-male (*F* = 6.244, *P *= 0.015). This interaction has distinct clinical implications: female exhibited positive correlations between sFC strength in this pathway and symptom severity, whereas male showed reduced sFC in the STG.R–MFG.L pathway compared to HC-male. In male, sFC strength in this circuit was negatively correlated with depressive symptoms, consistent with sensorimotor compensation probably driven by depressive symptom-induced disruption of emotional processing.

Network-level and molecular observations aligned with these regional findings: females displayed widespread ALFF abnormalities across multiple networks, whereas alterations in males were more focal. Molecularly, previous research has demonstrated that ALFF in the PCL.L was negatively correlated with depressive symptoms in female. Our findings show that ALFF in the PCL.L was positively associated with visual–spatial skills in males (Sun *et al*., 2022). This warrants further investigation but supports the notion of sex-specific neurofunctional alterations. Behaviourally, transcranial stimulation of the STG.R alters food preferences along sex lines (low-calorie preference in males vs. high-calorie preference in females) (Tu *et al*., 2022), providing external evidence for sex-specific functional roles of this region, which may be relevant to understanding its involvement in LLD.

Collectively, these findings demonstrate that sex differences in LLD are associated with distinct patterns of static neurofunctional alterations, particularly involving the PCL.L, the STG.R, and the sFC between the STG.R and MFG.L. The sFC of STG.R–MFG.L has emerged as a critical node showing significant disease‒sex interactions and divergent clinical correlations.

### Limitations

First, the cross-sectional design precludes causal inferences regarding the relationship between FC and cognitive/affective functions in LLD. Longitudinal studies are currently underway to elucidate the roles of ALFF, ReHo, and FC in LLD pathophysiology. Second, potential confounding effects of psychotropic medications were not controlled for in this analysis. Third, while the sample size provided adequate statistical power to detect primary effects, it limited sensitivity for identifying subtle sex differences and conducting comprehensive subgroup analyses. Future large-scale cohort studies are warranted to validate these findings and explore nuanced phenotypic variations. Fourth, the potential influence of symptom severity may mask partial sex-specific effects. Finally, we chose the altered brain region identified by ReHo and ALFF analysis to perform seed-based functional connectivity analysis. This approach may cause redundancy bias.

## Conclusion

This study revealed significant sex differences in the clinical manifestations and neurofunctional activity of LLD patients, with static metrics (ReHo and ALFF) showing robust group differences and diagnosis-specific interactions. Pronounced sex dimorphisms were observed in cognitive domains (memory, processing speed, executive function, and language) and in regional spontaneous activity (quantified by static ReHo and ALFF). Sex also moderated the links between right superior temporal gyrus activity and cognition and between left paracentral lobule activity and depressive symptoms. These findings underscore that LLD sex differences stem from stable static neurofunctional alterations (e.g. ReHo and ALFF), underpinning divergent clinical profiles. The lack of representation of dynamic effects suggests that static metrics better capture sex-specific mechanisms, offering reliable substrates for understanding heterogeneity. These static differences also hold potential as biomarkers for personalized diagnosis and intervention, bridging neurobiology and clinical phenotypes.

## Supplementary Material

kkaf029_Supplemental_File
