# Supplementary material for: Sex differences in brain activity and connectivity in late-life depression
Source: Psychoradiology. 2025 Dec 1;6:kkaf029. doi: 10.1093/psyrad/kkaf029 (PMC12892001; doi:10.1093/psyrad/kkaf029)
Supplement: kkaf029_Supplemental_File [file kkaf029_supplemental_file.docx]

The specific inclusion and exclusion criteria of particpants.

Inclusion Criteria:​​

Late-life Depression (LLD) Group:​​

1. Aged ≥ 55 years;
2. Met the DSM-IV diagnostic criteria for major depressive disorder (MDD);
3. Able to understand and complete questionnaire assessments;
4. Voluntarily agreed to participate in the study and undergo MRI scanning.

Healthy Control (HC) Group: Healthy participants matched with the LLD group in terms of gender, age, and educational level.

Exclusion Criteria:​​

1. Comorbid psychiatric disorders, including schizophrenia, bipolar disorder, bulimia/anorexia nervosa, or other mental disorders;
2. Neurological conditions, such as Parkinson’s disease, frontotemporal dementia, or Lewy body dementia;
3. History of stroke, cerebral hemorrhage, traumatic brain injury, or epilepsy;
4. Received electroconvulsive therapy (ECT) within the past month;
5. Presence of contraindications to MRI or structural brain abnormalities (e.g., brain tumors, cysts) detected via MRI

Table 1**.** ALFF of Left Paracentral Lobule，ReHo of Right Superior Temporal Gyrus and sFC in subjects

|  | LLD | | HC | | *P* | diagnosis statu  F(*P*) | sex statu  F(*P*) | diagnosis*sex  F(*P*) |
| --- | --- | --- | --- | --- | --- | --- | --- | --- |
|  | Male(n=27) | Female(n=48) | Male(n=55) | Female(n=109) |  |  |  |  |
| ALFF of Left Paracentral Lobule | -0.06±0.03 | -0.06±0.04 | -0.08±0.06 | -0.06±0.04 | **0.001**^a^** | 0.303(0.583)^c^ | **9.317(0.003**)**^c^ | **11.086(0.001**)**^c^ |
| ReHo of Right Superior Temporal | -0.22±0.32 | -0.07±0.32 | 0.05±0.35 | -0.24±0.34 | **<0.001***^a^** | 0.399(0.528)^c^ | 1.009(0.316)^c^ | **22.073(<0.001***)**^c^ |
| sFC between Right Superior Temporal gyrus and Left Middle Frontal | 0.47±0.12 | 0.51±0.12 | 0.56±0.15 | 0.48±0.13 | **0.001**^a^** | 2.63(0.106)^c^ | 0.864(0.353)^c^ | **10.994(0.001**)**^c^ |

When controlled age, years of education, 4 groups (LLD-female, LLD-male, HC-female and HC-male) were compared using **general liner model** (multivariate), showed that there were significant differences in diagnosis*sex status of ALFF, ReHo and sFC, besides there was significant difference of ALFF in sex status.

a One - way ANOVA, with data expressed as mean ± standard deviation.

b Kruska - Wallis test, with data expressed as median (interquartile range)

c general linear model

Abbreviations:

ALFF, Amplitude of low-frequency fluctuations; ReHo,regional homogeneity; sFC, static functional connectivity.

**P* < 0.05, ***P* < 0.01, ****P* < 0.001

Table 2. The interactive effect value of dynamic ALFF and dynamic ReHo by using 30 windowsize ( diagnosis vs. sex ).

|  | brain region | Peak MNI | | | peak intensity |
| --- | --- | --- | --- | --- | --- |
|  |  | X | Y | Z |  |
| ALFF | - |  |  |  |  |
| ReHo | Frontal_Inf_Orb_R | 45 | 21 | -15 | 16.7466 |
|  | Precentral_L | -39 | -15 | 66 | 17.4318 |

MNI, Montreal Neurological Institute; X, Y, Z are the coordinates of primary peak locations in the MNI space; *P*< 0.05, GRF-corrected. We regressed age, years of education and head motion as covariance by using interacted analysis.

Table 3. The interactive effect value of dynamic ALFF and dynamic ReHo by using 50 windowsize ( diagnosis vs. sex ).

|  | brain region | Peak MNI | | | Peak intensity |
| --- | --- | --- | --- | --- | --- |
|  |  | X | Y | Z |  |
| ALFF | Amygdala_R | 27 | 0 | -18 | 21.7893 |
|  | Frontal_Sup_L | -15 | 36 | 51 | 14.227 |
| ReHo | Precuneus_R | 3 | -60 | 27 | 16.1014 |
|  | Cingulum_Mid_L | -6 | -42 | 45 | 14.0853 |
|  | Supp_Motor_Area_R | 9 | 24 | 57 | 17.6483 |
|  | Frontal_Mid_L | -33 | 18 | 60 | 23.5326 |

MNI, Montreal Neurological Institute; X, Y, Z are the coordinates of primary peak locations in the MNI space; *P*< 0.05, GRF-corrected. We regressed age, years of education and head motion as covariance by using interacted analysis.

Table 4. The interactive effect value of dynamic ALFF and dynamic ReHo by using 70 Windowsize ( diagnosis vs. sex ).

|  | brain region | Peak MNI | | | peak intensity |
| --- | --- | --- | --- | --- | --- |
|  |  | X | Y | Z |  |
| ALFF | - |  |  |  |  |
| ReHo | Frontal_Mid_L | -33 | 18 | 60 | 15.1018 |

MNI, Montreal Neurological Institute; X, Y, Z are the coordinates of primary peak locations in the MNI space; *P*< 0.05, GRF-corrected. We regressed age, years of education and head motion as covariance by using interacted analysis.

Table 5. Partial correlation of ReHo (Right Superior Temporal Gyrus, STG.R) with HAMD-17 factors, ALFF (Left Paracentral Lobule, PCL.L) with cognitive functions and sFC (Right Superior Temporal Gyrus-Left Middle Frontal) with HAMD-17 factors in LLD-male and LLD-female

|  | Female  r(*P*) | Male  r(*P*) |
| --- | --- | --- |
| **ReHo of STG.R with HAMD-17 factors** | |  |
| Retardation | 0.201(0.47) | 0.065(1) |
| Cognitive impairment | 0.155(0.39) | -0.132(0.8) |
| Anxiety/somatization | 0.188(0.36) | -0.045(0.76) |
| Sleep disturbance | 0.083(0.59) | 0.25(1) |
| Weight | 0.392(**0.04***) | -0.207(0.43) |
| **ALFF of PCL.L with cognitive function** | |  |
| MMSE | 0.099(0.74) | 0.285(0.74) |
| AVLT | 0.16(0.67) | 0.397(0.43) |
| WMT | -0.011(0.93) | 0.11(0.82) |
| SDMT (s) | 0.04(0.85) | 0.117(1) |
| Stroop A (s) | -0.112(0.76) | 0.112(092) |
| TMT A | 0.133(0.73) | 0.023(0.93) |
| TMT B | 0.069(0.85) | 0.185(1) |
| Stroop B (s) | -0.056(0.83) | 0.129(1) |
| BNT | 0.233(0.48) | 0.405(0.39) |
| VFTJS2 | 0.294(0.32) | -0.101(0.77) |
| ROCF | 0.223(0.38) | 0.666(**0.044***) |
| **STG.R-based sFC with HAMD-17 factors** | |  |
| Retardation | 0.203(0.3) | 0.081(0.89) |
| Cognitive impairment | 0.189(0.27) | -0.151(0.8) |
| Anxiety/somatization | 0.265(0.2) | -0.026(0.91) |
| Sleep disturbance | 0.083(0.59) | 0.31(0.7) |
| Weight | 0.436(**0.02***) | -0.282(0.45) |

Abbreviations:

MMSE, mini-mental state examination; AVLT(N4), short delay free recall of auditory verbal learning test; WMT, Working Memory Test;

SDMT, symbol digit modalities test; Stroop, Stroop Color and Word Test; TMT, trail making test; BNT, Boston naming test; VFT, verbal fluency test; ROCF, Rey-Osterrieth Complex Figure；LLD, Late-life depression; HCs, healthy controls; HAMD-17, 17-item Hamilton Depression Rating Scale; ALFF, Amplitude of low-frequency fluctuations; ReHo,regional homogeneity; sFC, static functional connectivity.

*P < 0.05, **P < 0.01, ***P < 0.001

Table 6. Post-hoc analysis of moderation effect when outcome is MMSE .

|  | Model 1 | Model 2 | Model 3 |
| --- | --- | --- | --- |
| Constant | 8.201 (2.284) | 6.919*(2.149) | 7.366*(2.359) |
| age | 0.455718 | 0.350784 | 0.408968 |
| study | 0.197*(2.029) | 0.271*(3.062) | 0.253*(2.947) |
| Head motion | 0.919 (0.102) | -0.486 (-0.060) | 1.964 (0.249) |
| ReHo | 0.114 (0.107) | -0.883 (-0.901) | 0.736 (0.629) |
| Sex (male) |  | 12.392068 | -3.015(-4.711) |
| ReHo*Sex |  |  | 10.95888 |
| *N* | 75 | 75 | 75 |
| R² | 0.175 | 0.352 | 0.402 |
| Adjust R² | 0.128 | 0.305 | 0.349 |
| F | F(4,70) = 3.715,  *P* = 0.008 | F(5,69) = 7.500, *P* < 0.001 | F(6,68) = 7.604,  *P* < 0.001 |
| △R² | 0.175 | 0.177 | 0.049 |
| △F | F(4,70)=3.715,  *P* = 0.008 | F(1,69)=18.851,  *P* < 0.001 | F(1,68)=5.615,  *P* = 0.021 |

Table 7. Post-hoc analysis of moderation effect when outcome is Weight (HAMD-17 factor).

|  | Model 1 | Model 2 | Model 3 |
| --- | --- | --- | --- |
| Constant | -0.068 (-0.124) | 0.063 (0.121) | -0.159 (-0.311) |
| age | 0.002(0.238) | -0.001 (-0.183) | 0.002 (0.235) |
| study | -0.003 (-0.179) | -0.009 (-0.632) | -0.007 (-0.520) |
| Head motion | 0.186(0.137) | 0.513(0.400) | 0.869(0.698) |
| ALFF | 0.061(0.369) | 0.204(1.251) | -0.061(-0.321) |
| Sex (male) |  | 0.332**(3.064) | 0.384*(3.605) |
| ALFF*Sex |  |  | 0.863*(2.477) |
| N | 75 | 75 | 75 |
| R² | 0.004 | 0.124 | 0.196 |
| Adjust R² | -0.053 | 0.06 | 0.125 |
| F | F(4,70) = 0.076,  P = 0.989 | F(5,69) = 1.945,  P= 0.098 | F(6,68) = 2.765,  P = 0.018 |
| △R² | 0.004 | 0.119 | 0.073 |
| △F | F(4,70) = 0.076,  *P* = 0.989 | F(1,69) = 9.388,  *P* = 0.003 | F(1,68) = 6.137,  *P* = 0.016 |

Table 8. Post-hoc analysis of moderation effect when outcome is Weight (HAMD-17 factor).

|  | Model 1 | Model 2 | Model 3 |
| --- | --- | --- | --- |
| Constant | -0.820(-1.141) | -0.792(-1.093) | -0.720(-1.024) |
| age | 0.029(1.474) | 0.027(1.344) | 0.022(1.127) |
| study | 0.013(1.291) | 0.012(1.218) | 0.011(1.146) |
| Head motion | -1.220(-0.669) | -1.176(-0.641) | -0.778(-0.435) |
| sFC | 0.866(1.485) | 0.919(1.542) | 1.949**(2.679) |
| Sex (male) |  | 0.073(0.496) | 0.051(0.358) |
| FC*Sex |  |  | 6.27935 |
| *N* | 75 | 75 | 75 |
| R² | 0.078 | 0.081 | 0.149 |
| Adjust R² | 0.025 | 0.014 | 0.074 |
| F | *F* (4,70) = 1.472,  *P* = 0.220 | *F* (5,69) = 1.214,  *P* = 0.312 | *F* (6,68) = 1.981,  *P* = 0.080 |
| △R² | 0.078 | 0.003 | 0.068 |
| △F | *F* (4,70)=1.472,  *P* = 0.220 | *F* (1,69) = 0.246,  *P* = 0.622 | *F* (1,68) = 5.428,  *P* = 0.023 |

Table 9. Medication of patient in LLD.

|  | Medication |
| --- | --- |
| 0 | unmedicated |
| 1 | SSRI |
| 2 | SNRI |
| 3 | NaSSA |
| 4 | TCA |
| 5 | 5-HT2C or 5-HT1A |
| 6 | SSRI + NaSSA |
| 7 | SSRI + 5-HT1A/5-HT2C |
| 8 | SNRI + 5-HT1A/5-HT2C |
| 9 | SNRI + NaSSA + 5-HT1A |

Table 10. Partial correlation of ReHo with HAMD-17 factors, ALFF with cognitive functions and sFC with HAMD-17 factors in LLD-male and LLD-female （covariance：illness duration(month), medication, age and years of education).

|  | Female  r(p) | Male  r(p) |
| --- | --- | --- |
| **ReHo of STG.R with hamd-17 factors** |  |  |
| Retardation | 0.159(0.31) | 0.133(0.839) |
| Cognitive impairment | 0.145(0.355) | 0.1(0.713) |
| Anxiety/somatization | 0.195(0.21) | -0.023(0.933) |
| Sleep disturbance | 0.129(0.408) | -0.055(0.839) |
| Weight | **0.346(0.023^*^)** | -0.062(0.819) |
| **ALFF of PCL.L with cognitive function** |  |  |
| MMSE | 0.01(0.955) | 0.488(0.107) |
| AVLT | -0.13(0.465) | **0.639(0.025^*^)** |
| WMT | 0.036(0.841) | 0.786(0.002) |
| SDMT (s) | 0.085(0.632) | 0.307(0.331) |
| Stroop A (s) | 0.228(0.194) | 0.114(0.726) |
| TMT A | 0.25(0.154) | 0.202(0.529) |
| TMT B | 0.07(0.692) | 0.319(0.313) |
| Stroop B (s) | -0.207(0.239） | 0.137（0.672） |
| BNT | 0.286（0.102） | 0.569（0.053） |
| VFTJS2 | 0.275（0.116） | ‘0.059（0.856） |
| ROCF | 0.115（0.516） | **0.655（0.021^*^）** |
| **ReHo-based sFC with hamd-17 factors** |  |  |
| Retardation | 0.154(0.323) | 0.046(0.865) |
| Cognitive impairment | 0.175(0.263) | 0.004(0.987) |
| Anxiety/somatization | 0.249(0.108) | -0.007)0.979) |
| Sleep disturbance | 0.119(0.447) | -0.122(0.654) |
| Weight | **0.419(0.005^*^)** | -0.163(0.546) |

Table 11. LLD-male, LLD-female, HC-male and HC-female performed on Kruskal-Wallis (K-W) test.

|  | LLD | | HC | | *P*  (K-W test) | *P*  (ANOVA) |
| --- | --- | --- | --- | --- | --- | --- |
|  | Male (n=27) | Female (n=48) | Male (n=55) | Female (n=109) |  |  |
| Age (years) | 67(10) | 65(7) | 68(11) | 67(8) | 0.42 | 0.424 |
| Years of education | 9(4) | 9(5.75) | 10(5) | 10.5(3.75) | 0.148 | 0.096 |
| **Cognitive functions** | | | | | | |
| **Global cognitive function** | | | | | | |
| MMSE | 25.5(5) | 26(4) | 27(3) | 27(3) | **0.002*** | <**0.001***** |
| Memory |  |  |  |  |  |  |
| AVLT (N4) | 5(4) | 6(3) | 5.5(4) | 7(3) | **0.03*** | <**0.001***** |
| WMT | 4(3) | 5(4) | 6(4) | 6(4) | **0.027*** | **0.03*** |
| **Information processing speed** | | | | | | |
| SDMT (s) | 29(11) | 27(20) | 32(17) | 35(14) | <**0.001***** | <**0.001***** |
| TMT A | 57(27) | 67(34) | 47(28) | 48(25) | **0.006*** | **0.005**** |
| Stroop A (s) | 33(10) | 31(8) | 30（12） | 27（8） | **0.001**** | **0.003**** |
| **Executive function** | | | | | | |
| TMT B | 71(14) | 77(44) | 65.5(39) | 63(40) | 0.325 | 0.47 |
| Stroop B (s) | 47.5(16) | 40(14) | 43.5(21) | 37(11) | **0.007*** | **0.001**** |
| **Language** | | | | | | |
| BNT | 22(5) | 20(3) | 23(5) | 21(4) | <**0.001***** | <**0.001***** |
| VFT | 12(2.75) | 13(6) | 13(5.25) | 14(5.5) | **0.019*** | **0.012*** |
| **Visuospatial skill** | | | | | | |
| ROCF | 8(3.88) | 7(9.5) | 11.25(8.5) | 10.5(9) | **0.015*** | **0.005**** |
| **HAMD-17 factors** | | | | | | |
| Retardation | 2(4) | 2(4) | 0(0) | 0(0) | <**0.001***** | <**0.001***** |
| Cognitive bias | 1(2) | 0(1.75) | 0(0) | 0(0) | <**0.001***** | <**0.001***** |
| anxiety/somatization | 2(3) | 3(5) | 0(1) | 0(2) | <**0.001***** | <**0.001***** |
| Sleep disturbance | 2(4) | 2(3) | 0(1) | 0(2) | <**0.001***** | <**0.001***** |
| Weight | 0(0) | 0(0) | 0(0) | 0(0) | <**0.001***** | <**0.001***** |
| total score | 7(11) | 7.5(12) | 1(3) | 1(3) | <**0.001***** | <**0.001***** |

Figure 1. Post-hoc analysis of Moderation effect.


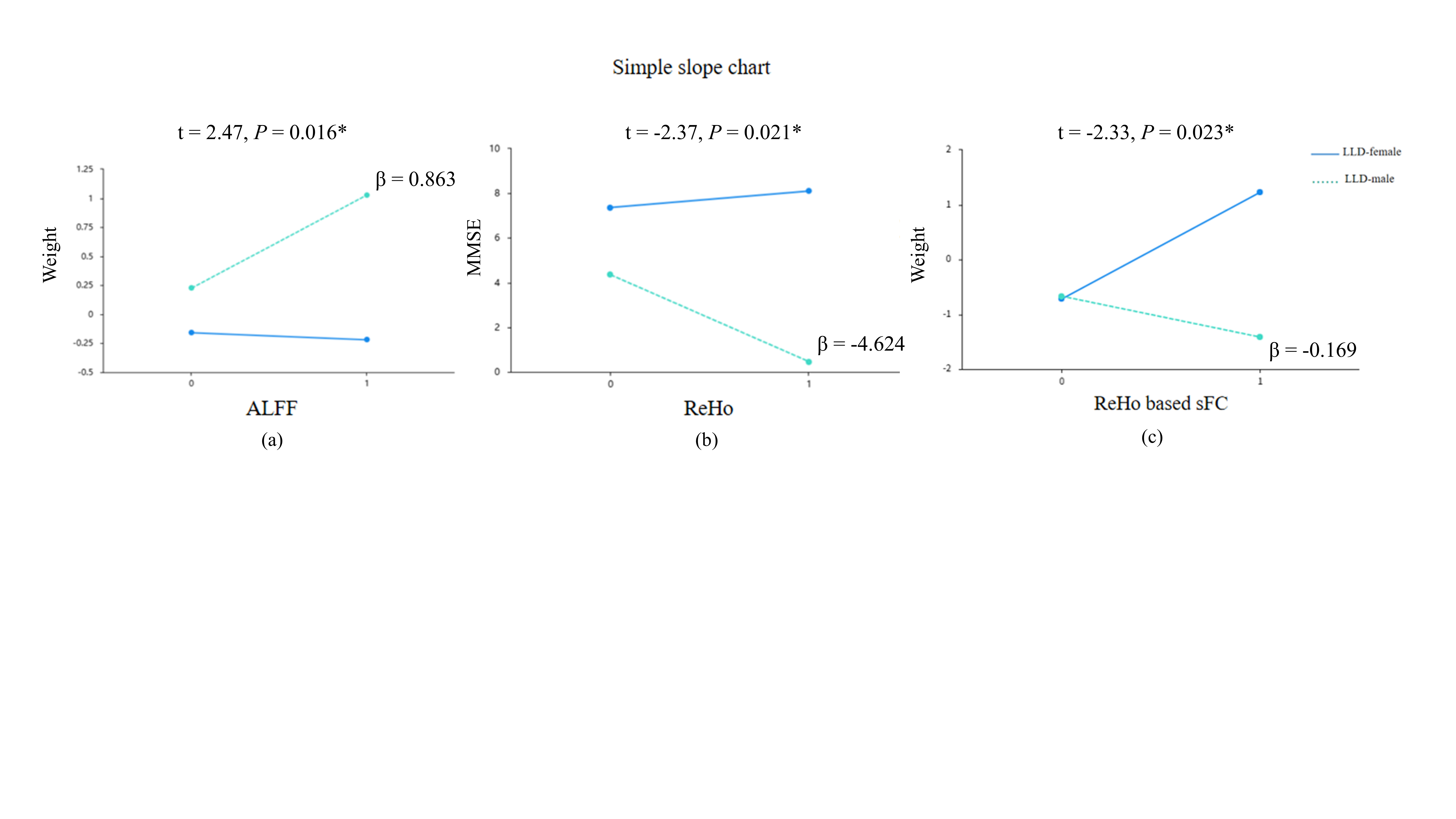


Figure 2. Moderating effects of sex on the regional brain activity and cognition, depressive symptom in LLD patients.


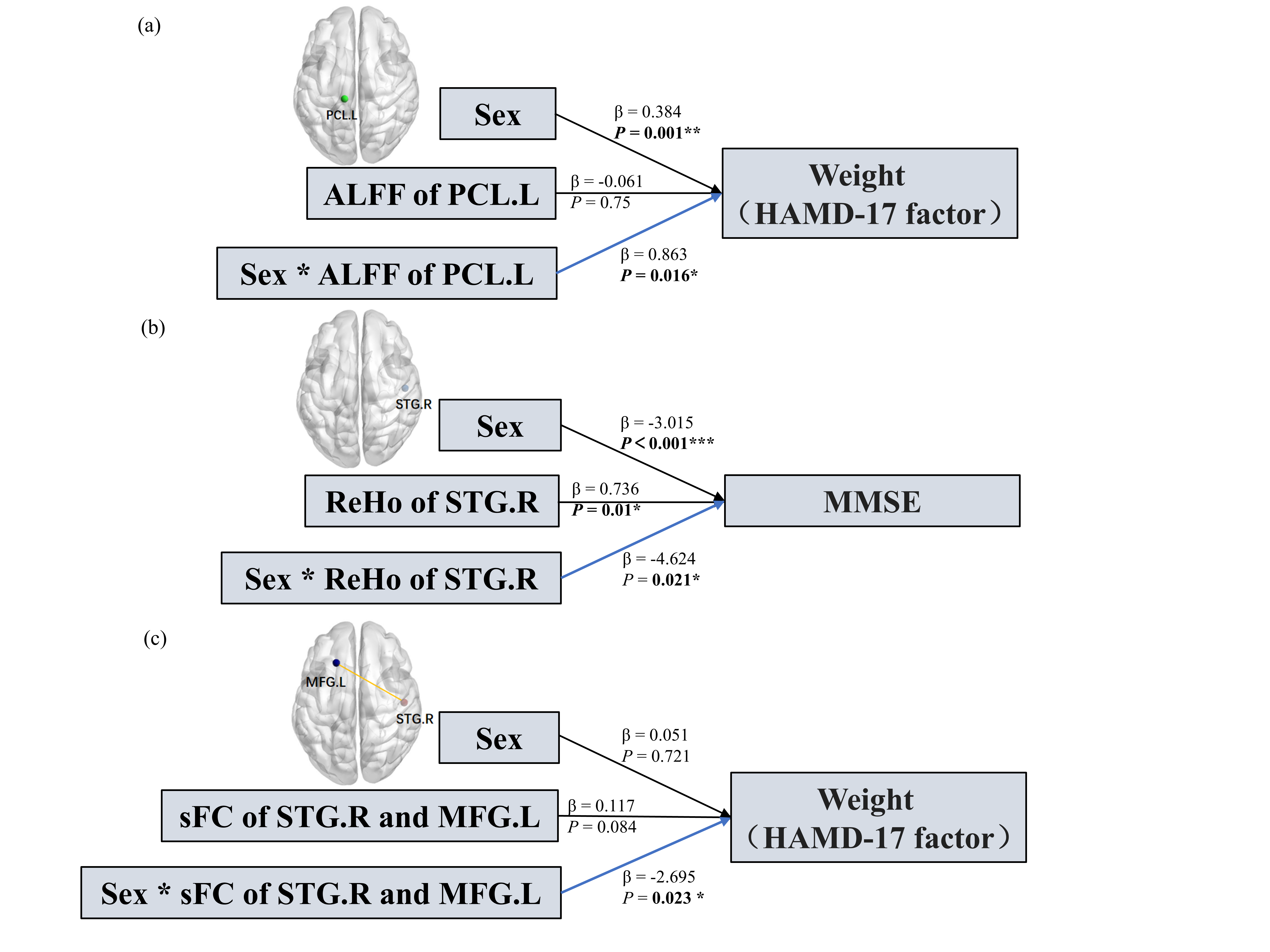


The mediation model was calculated in SPSS.26 process. Non-significant differences were defined as P > 0.05.

The moderation analysis was performed to investigate the relationship between ALFF (left paracentral lobule) in LLD (predictor), depressive symptoms (outcome), ReHo (right superior temporal gyrus) in LLD (predictor), cognitive scores (outcome), sFC (right superior temporal gyrus - Left Middle Frontal) in LLD (predictor), depressive symptoms(outcome) and sex (mediator), while controlling age, years of education and head motion as covariance.

Abbreviations:

HAMD-17, 17-item Hamilton Depression Rating Scale; MMSE, mini-mental state examination; ALFF, amplitude of low-frequency fluctuations; ReHo, regional homogeneity; sFC, static functional connectivity; PCL.L, left paracentral lobule; STG.R, right superior temporal gyrus; MFG.L, left middle frontal gyrus.

*P < 0.05, **P < 0.01, ***P < 0.001
